# Hospital Inpatient versus HOme-based rehabilitation after knee arthroplasty (The HIHO study): study protocol for a randomized controlled trial

**DOI:** 10.1186/1745-6215-14-432

**Published:** 2013-12-17

**Authors:** Mark A Buhagiar, Justine M Naylor, Ian A Harris, Wei Xuan, Friedbert Kohler, Rachael J Wright, Renee Fortunato

**Affiliations:** 1 Braeside Hospital, HammondCare, Locked Bag 82, Wetherill Park 2164, NSW, Australia; 2 South West Sydney Clinical School, University of New South Wales, Liverpool Hospital, Elizabeth Drive, Liverpool, NSW 2170, Australia; 3 South West Sydney Local Health District, Locked Bag 7103, Liverpool 2170NSW, Australia; 4 Whitlam Orthopaedic Research Centre, PO Box 906, Caringbah, NSW 2229, Australia; 5 5Ingham Institute of Applied Medical Research, PO Box 3151Westfields Liverpool, Liverpool 2170NSW, Australia

## Abstract

**Background:**

Formal rehabilitation programs are often assumed to be required after total knee arthroplasty to optimize patient recovery. Inpatient rehabilitation is a costly rehabilitation option after total knee arthroplasty and, in Australia, is utilized most frequently for privately insured patients. With the exception of comparisons with domiciliary services, no randomized trial has compared inpatient rehabilitation to any outpatient based program. The Hospital Inpatient versus HOme (HIHO) study primarily aims to determine whether 10 days of post-acute inpatient rehabilitation followed by a hybrid home program provides superior recovery of functional mobility on the 6-minute walk test (6MWT) compared to a hybrid home program alone following total knee arthroplasty. Secondarily, the trial aims to determine whether inpatient rehabilitation yields superior recovery in patient-reported function.

**Methods/Design:**

This is a two-arm parallel randomized controlled trial (RCT), with a third, non-randomized, observational group. One hundred and forty eligible, consenting participants who have undergone a primary total knee arthroplasty at a high-volume joint replacement center will be randomly allocated when cleared for discharge from acute care to either 10 days of inpatient rehabilitation followed by usual care (a 6-week hybrid home program) or to usual care. Seventy participants in each group (140 in total) will provide 80% power at a significance level of 5% to detect an increase in walking capacity from 400 m to 460 m between the Home and Inpatient groups, respectively, in the 6MWT at 6 months post-surgery, assuming a SD of 120 m and a drop-out rate of <10%.

The outcome assessor will assess participants at 10, 26 and 52 weeks post-operatively, and will remain blind to group allocation for the duration of the study, as will the statistician. Participant preference for rehabilitation mode stated prior to randomization will be accounted for in the analysis together with any baseline differences in potentially confounding characteristics as required.

**Discussion:**

The HIHO Trial will be the first RCT to investigate the efficacy of inpatient rehabilitation compared to any outpatient alternative following total knee arthroplasty.

**Trial registration:**

U.S. National Institutes of Health Clinical Trials Registry (http://clinicaltrials.gov) ref: NCT01583153

## Background

In Australia, inpatient rehabilitation is a costly and commonly utilized treatment option after total knee arthroplasty (TKA), particularly in the private sector. A 12-day inpatient stay in a private facility costs approximately AU$8,100 [1]. Based on unpublished data derived from both the Australian Rehabilitation Outcomes Centre and the National Joint Replacement Registry, approximately 46% of privately insured patients undergoing knee replacement (primary, revision or unicompartmental) in New South Wales, and 32% of private patients Australia-wide, received inpatient rehabilitation in the 2011/12 financial year. As the majority of TKA procedures are performed in the private sector in Australia - some 32,105 per year [2] - the question of whether inpatient rehabilitation yields superior outcomes to cheaper alternatives is of considerable interest to private health insurers and governments alike. Inevitably, the cost of inpatient services is reflected in private insurance premiums and costly premiums negatively affect rates of private health insurance.

### Total knee arthroplasty and associated costs

Between 2003 and 2012, the number of primary TKA procedures undertaken in Australia has increased by 83.7% [2]. This increasing surgical volume is consistent with international trends [3-6]. Demand is increasing due to advances in surgical technique [7], the ageing population and, thus, more people with osteoarthritis (OA), and because consumers are opting for a surgical solution determined to reduce age-related disability in an effort to maintain health and standards of living as they live longer [8,9]. Further increases are expected [2]; thus, there is growing concern regarding the sustainability and affordability of this type of intervention both in the public and private sectors. Increased demand in the public sector cannot be readily met because the supply of services is not driven by demand, but rather is dictated by governmental policy set within the context of a fixed proportion of gross domestic product [8]. Whilst the private sector can adjust more readily to increases in demand, this inevitably puts upward pressure on private health insurance premiums [7]. Whilst the TKA procedure is viewed as highly cost effective in light of the impressive gains in functional performance and health-related quality of life [10,11], the acute care and associated rehabilitative costs impose a significant burden on public and private hospital budgets and resources [12-14]. Not surprisingly, a recent retrospective study from the US concluded that the cost effectiveness of TKA is reduced if the procedure is associated with a stay in an inpatient rehabilitation facility [10].

### Evidence in support of inpatient rehabilitation after total knee arthroplasty

A systematic search of MEDLINE, EMBASE and CENTRAL healthcare databases - first completed in October 2011 and then again in July 2013 (see Table 1 for search strings) - revealed that there was no high level evidence to support the provision of inpatient rehabilitation after TKA over any outpatient-based alternative. Specifically, no randomized trial has compared inpatient rehabilitation to any group-based or center-based one-to-one program or a monitored or unmonitored home program. Two studies (one review [15] and one randomized controlled trial (RCT) [16]) undertaken in the US have concluded that inpatient rehabilitation is not superior to domiciliary rehabilitation (hospital services provided at home). A non-randomized pilot study in Germany concluded that inpatient rehabilitation was not more cost effective compared to outpatient rehabilitation [17]. Longitudinal data from an Australian cohort [13] observed that patient-reported outcomes were similar whether or not TKA patients were discharged to inpatient rehabilitation. The authors concluded that randomized trials were required to explore who, if any group, benefits most (such as older or more infirm patients) from inpatient rehabilitation.

**Table 1:** Medline search string

**No**	**Searches**	**Results**
1	Exp rehabilitation/	147587
2	(rehabilit$ or habilitat$).mp.	115199
3	Exp physical therapy modalities/	121900
4	(Physical therap$ or physiotherap$).mp.	43406
5	1 or 2 or 3 or 4	315967
6	Arthroplasty, replacement, knee.mp.	12403
7	Knee prosthesis/	8491
8	Knee prosthesis/or arthroplasty, replacement, knee/or knee joint/	49918
9	Exp knee/	10214
10	Knee/or knee prosthesis/or athroplasty, replacement, knee/or knee joint/or osteoarthritis, knee/	63871
11	9 or 10	63871
12	Exp arthroplasty/	38632
13	Joint prosthesis/	8761
14	(Arthroplast$ or prosthe$ or replac$).mp.	493605
15	12 or 13 or 14	494353
16	11 and 15	21729
17	6 or 7 or 8 or 16	51109
18	Randomized controlled trial.pt.	380312
19	Controlled clinical trial.pt.	88731
20	Randomi?ed.ab.	332438
21	Placebo.ab.	149978
22	Randomly.ab.	194497
23	Clinical trials as topic.sh.	173251
24	Trial.ti.	120939
25	18 or 19 or 20 or 21 or 22 or 23 or 24	888705
26	Exp animals/not humans.sh.	4007869
27	25 not 26	819207
28	Meta analysis.mp,pt. or review.pt. or search$.tw.	2047494
29	27 or 28	2732075
30	5 and 17 and 29	1316
31	Limit 30 to english language	1212

### Evidence in support of non-inpatient modes of rehabilitation

A recent systematic Cochrane review concluded that there was insufficient and inconsistent evidence to recommend any specific type, timing or setting for post-acute TKA rehabilitation, and there was low therapeutic validity among trials related to exercise intensity, dosage and adherence (Westby M, 2013, personal communication). This included the use of hydrotherapy, one-to-one or group-based therapy, and even different exercise types such as those targeting functional movements versus isolated muscle activity. Notably, most of the studies reviewed were small (n ≤ 160) and none has simultaneously compared group-based, one-to-one and home-based programs. Our own recently completed, comparatively large RCT (n = 249) [18] provides strong evidence that a one-to-one outpatient program does not provide superior patient-reported outcomes compared to less resource-intensive modes such as group-based or monitored home programs up to 1 year post-surgery. In addition, similar recovery patterns were observed across all three study arms in joint range, quadriceps lag, and timed mobility. The similar recovery patterns were observed despite the fact that access to center-based interventions - 12 sessions each for either the one-to-one or group-based study arm - were optimized through the use of transport and parking concessions. Thus, lack of access or poor attendance did not explain lack of superiority of the one-to-one mode.

### Significance

Based on a recent RCT [18] and the lack of research evaluating the necessity of inpatient rehabilitation, we contend that a trial comparing the efficacy of the most resource-intensive form of rehabilitation delivery after TKA - inpatient rehabilitation compared to one with comparatively little resource use such as a monitored home program - is justified. The study will be a landmark study in this area, providing evidence that either supports or refutes the need for resource intensive inpatient rehabilitation after TKA.

### Primary objective

The primary objective of this study is to establish whether inpatient rehabilitation is beneficial after TKA for patients with OA who could otherwise be discharged directly home.

The main hypothesis to be tested by the proposed study is that TKA recipients who receive inpatient rehabilitation in addition to participating in a home program, compared to patients who participate in a home program only, will achieve a superior level of mobility at 6 months post-surgery as assessed by the 6-minute walk test (6MWT).

## Trial design and methods

### Recruitment and consent

We will recruit 140 participants through the Whitlam Joint Replacement Centre (WJRC) at Fairfield Hospital in New South Wales, Australia. Consecutive patients presenting for primary unilateral TKA will be screened for eligibility. Patients not participating in the study will receive usual care (a monitored home program).

To be eligible, participants must meet the following inclusion criteria:

• Undergoing primary, unilateral elective TKA

• Primary diagnosis of OA as documented in the medical record

• Willingness to give written informed consent and willingness to participate in and comply with the study.

• Age 40 years or over

Exclusion criteria will include:

• A history of a mental illness or a condition which would interfere with the patient’s ability to understand the requirements of the study. This may include, but is not limited to, a history of dementia or short-term memory impairment secondary to a cerebrovascular accident.

• A predisposition to be discharged to an inpatient rehabilitation (or hostel) facility due to lack of social support (for example, the patient would otherwise return home with no carer availability) or other physical impairments (for example, contra lateral amputated lower limb).

• Patients unable to read English

• Patients unable to perform a home exercise program without hands-on support from another person or who are unable to perform the program without supervision (that is, observation) from another person.

• Patients restricted to partial or no weight-bearing through the operated limb post-surgery

• Patients experiencing a complication post-surgery which precludes participation in the planned rehabilitation programs (for example, they suffer a stroke or peri prosthetic fracture).

• Pregnancy.

**Figure 1:**
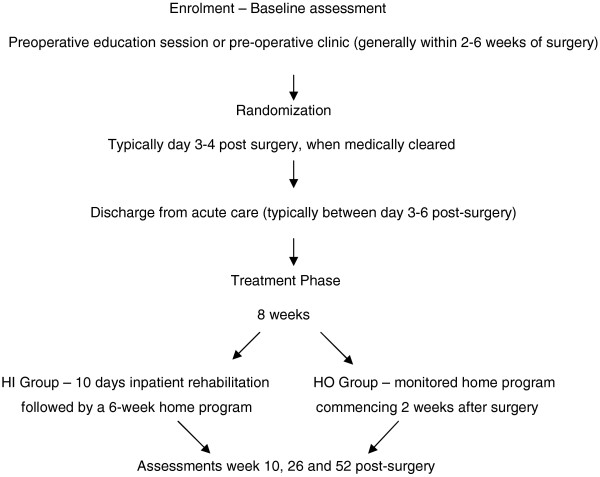
**Cohort ascertainment, randomization and study timeline.** HI, hospital inpatient rehabilitation; HO, hybrid home program.

Figure 1 shows the expected flow of participants through recruitment, assessment, intervention and follow-up. The co-ordinating investigator (MAB) will identify potential participants presenting to the WJRC orthopedic clinic, screening consecutive patients against the inclusion and exclusion criteria by chart review and direct questioning. A screening log will be kept, recording the criteria eliminating all those found to be ineligible. A second log will be maintained, detailing reasons for unwillingness to consent to participation in the study by otherwise eligible patients. Those willing to participate will be asked for informed consent using an approved consent form and baseline measures will be obtained.

This trial is a two-arm parallel RCT (Figure 1). The two study groups are: (1) hospital inpatient (HI) and (2) home-based group (hybrid home program (HO)).

Potential participants who are unwilling to undergo randomization will be invited to participate in a third, non-randomized, observational group, who will be followed up 6 months after surgery. This third group will permit the detection of a possible preference effect amongst recruited participants. If study recruitment is biased towards people with a preference for inpatient rehabilitation, there is a risk that participants randomized to the inpatient arm will, in part, report superior outcomes compared to those randomized to the home program because they received their preferred program. If no such superiority is observed when we compare those who were randomly allocated to usual care and preferred inpatient rehabilitation to those who opted to receive usual care (those in the observational arm), then it is possible that any preference effect (if present) is not strong.

The study is expected to take up to 51 months, consisting of 30 to 33 months for recruitment and randomization, 12 months for follow-up of the final participants, and 6 months for analysis.

### Randomization and allocation concealment

Randomization will take place following surgery once it has been confirmed that participants are likely to be cleared for discharge home by post-operative day 5. If it is determined that there is a need for rehabilitation at this time - owing to a post-operative complication - such patients will not be eligible to participate in the study.

A centralized randomization service will be used for participant randomization using a 1:1 ratio, providing secure, coded randomization via telephone. Group allocation, therefore, will be concealed from all parties until the result of the randomization is known. Participants will be randomized to one of the two intervention groups using the method of minimization stratified for variables that affect the primary outcome (distance walked in the 6MWT), age (≤68 years, >68 years), height (≤163 cm, >163 cm), and gender [19].

### Blinding

Outcome assessors will be blind to group allocation, and will not be involved in providing the interventions. The physiotherapists delivering the intervention cannot be blinded to intervention, but will not play a part in the collection or analysis of outcomes, and will only provide one of the treatment options. Those performing the statistical analyses will be blind to group allocation until the evaluation is completed.

### Interventions

Protocols for the two arms of the study will be described in a Standard Procedures Manual, available from the co-ordinating investigator. A guide for tailoring exercise prescription and advancement, as well as criteria for discontinuing allocated interventions, will be included within these protocols.

### Hospital inpatient rehabilitation

Those allocated to HI will be admitted to the adjacent rehabilitation hospital for 10 days. As per the private sector, HI participants will receive twice-daily supervised physiotherapy comprising 1 to 1.5 hour class-based exercises and 1 to 1.5 hour one-to-one therapy. Prior to discharge, participants will be familiarized with the home program as described below.

### Hybrid home program (standard care)

The HO will be based on what is standard care in the local health district, which is broadly based on the home program utilized in our most recent RCT [18]. The original home program was informed by guidelines for exercise in the elderly [20] and those with osteoarthritis [21]. Approximately 2 weeks post-surgery, participants allocated to the HO will attend one group-based outpatient exercise session in the physiotherapy department where the home program will be rehearsed and exercises individualized as required due to co-morbidities. The program comprises general aerobic components as well as general functional and muscle-specific exercises focused on restoring knee mobility, lower limb strength, and normal neuromuscular co-ordination and gait patterns.

Participants in both treatment arms will be encouraged to attend one to two classes from post-operative weeks 3 to 8 to encourage exercise progression and discuss any ongoing issues with the therapist. Participants will receive a booklet detailing the home program, and will be permitted to contact the therapist by phone in this period.

All participants will be required to complete a diary detailing program adherence, healthcare utilization, and social costs relating to carer-burden. Participant attendance at the outpatient classes will also be obtained from the treating physiotherapist.

### Outcome measures

The primary outcome and most secondary outcomes will be measured pre-operatively, at week 10 (the time when the home program formally ceases), and then at 6 and 12 months post-randomization (Table 2). Assessments will be performed by an observer blinded to the participants’ study allocation.

**Table 2:** Outcome measures

Primary outcome measure*	**Data collection instrument**
6 minute walk test*	6 minute walk test; physical test [19]
**Secondary outcome measures**	
Gait speed	15 m walk test; physical test
Knee injury and Osteoarthritis Outcome Score (KOOS)	KOOS [22]; patient-rated questionnaire
Oxford Knee Score (OKS)	OKS [23]; patient-rated questionnaire
EQ-5D-5 L	EQ-5D-5 L [23]; patient-rated questionnaire
Knee range of motion	<100 degrees, ≥100 degrees
Return to work	Self-recorded diary
Complication data	Self recorded diary; health system records
Healthcare consumption and related costs	Self recorded diary; health system records

*The primary end-point for data analysis is 6 months post-surgery. All outcome measures will be undertaken pre-surgery, then at 10, 26 and 52 weeks post-surgery, with the exception of return to work and healthcare consumption and related costs, which will not be assessed at baseline.

#### Primary

The primary outcome is walking distance at 6 months post-surgery measured using the 6MWT [24-26]. A functional outcome based on a physical test is a novel choice as a primary outcome in this field given that the convention with most TKA rehabilitation studies is to focus on patient-reported outcomes [16,25-28]. The decision to employ measured mobility as the primary outcome was based on multiple factors. First, functional mobility is a composite of several factors targeted in rehabilitation programs after TKA such as lower limb strength, knee range of motion, and balance [25,26,28-30]. Second, a functional outcome is more likely to be directly influenced by the intervention (rehabilitation), and the intervention aims to improve walking. Third, the 6MWT is highly reproducible within the individual [31]. Fourth, it is likely to be less susceptible to misinterpretation and less culturally sensitive than patient-reported outcomes. Fifth, the test does not appear to suffer from the floor or ceiling effects associated with many patient-reported outcomes [21]. Sixth, an observer-measured outcome is less likely to be influenced by a preference effect compared to a patient-reported outcome, and this is particularly important when the intervention under examination cannot be blinded from the recipient. Together, these attributes mean the results for our primary outcome should be readily translatable to any TKA cohort.

#### Secondary

Secondary outcomes comprise both patient-reported and observer-measured outcomes including a knee pain and function survey (the Knee injury and Osteoarthritis Outcome Score (KOOS) [22], knee range of motion (ROM) (<100 degrees, ≥100 degrees) [32,33], health-related quality of life (the EQ-5D) [34] and the proportion achieving a gait speed of 1.2 m/s during a 15 m walk test). Direct healthcare costs will also be captured using diarized recordings and face-to-face interviews [12,13,15]. Costs recorded will include visits to health professionals, pathology tests, imaging, cost of transport and cost of medication. Indirect costs including time lost from work for the patient or carer will also be explored in a secondary analysis. The extent to which a cost analysis is undertaken will depend on the availability of funds to perform a comprehensive health economic analysis.

### Baseline and other data

Demographic data (age, gender, height, weight, body mass index (BMI), level of education, co-morbidities) will be collected at baseline, and complication data (such as presentation to emergency department, re-admission, re-operation, knee manipulation, wound infection, venous thrombo-embolism and death) will be collected until 12 months post-surgery. Preference for rehabilitation mode (home-based rehabilitation program, inpatient rehabilitation program or either) will be ascertained prior to surgery and after formal rehabilitation has ceased [35].

### Follow-up

A summary of the follow-up schedule is shown in Figure 1. Researchers will perform assessments at baseline, 10 weeks, 6 months and 1 year. The co-ordinating investigator will call each participant within the week preceding each of their follow-up appointments to promote participant retention and completion of follow-up.

### Sample size

The primary end-point in this trial is functional mobility at 6 months post-surgery measured using the distance walked during a 6MWT. Seventy participants in each group (140 in total) will provide 80% power at a significance level of 5% to detect an increase in walking capacity from 400 m to 460 m between the HO and HI groups, respectively, in the 6MWT at 6 months post-surgery, assuming a SD of 120 m and a drop-out rate of <10%. The SD of 120 is a conservative estimate of the SD of the mean 6MWT at 6 months [18,25]. Using a 0.5SD criterion, this sample will be powered to detect a moderate effect size of 60 m difference in walking distance. If the actual SD of the sample collected is smaller than the assumed value, say 100 instead of 120, the study will remain powered to detect a moderate effect size of 0.5SD.

The original plan for the primary outcome was gait speed (m/s) at 6 months post-surgery, with an initial sample size powered to detect an absolute difference of 20% in the proportion of participants at 6 months who achieve the minimum gait speed considered necessary to cross safely at a pedestrian crossing (that is, 1.2 m/s). In the absence of other context-specific data, the data used for this calculation was based on the proportion of TKA participants (37%) who achieved this average gait speed at 6 months post-surgery during a 6MWT having participated in outpatient-based rehabilitation only [18]. This calculation assumed that average gait speed during a 6MWT was representative of the average speed a TKA recipient would achieve during a shorter test, such as crossing a 4- to 6-lane road (approximately 15 m). Aware that the assumption may be overly optimistic, we planned to review - whilst blind to treatment allocation - the proportion in each group achieving an average gait speed of 1.2 m/s during the 15 m walk test after the first 15 randomized participants reached the 10-week follow-up. On review, approximately 70% in each group achieved the requisite gait speed; hence, to expect inpatient rehabilitation to secure this gait speed for over 90% of the participants was deemed unrealistic. The primary outcome remained based around the 6MWT as originally planned, but was converted to the distance walked during this same test. To complement the revised primary outcome, and in the absence of data describing the minimum clinically important difference for the 6MWT, we included a sub-study to evaluate the minimum clinically important difference for measured mobility in this population.

### Data management and analysis

#### Data management

Data will be sent by blinded assessors to blinded data entry staff for collation and data entry, with range checks for data values. All databases will be password protected and stored in secure areas.

#### Descriptive analyses at baseline

Comparability of intervention groups will be investigated at baseline. Descriptive statistics will be presented, including summary statistics of potential confounding variables.

#### Analyses subsets

Data analysis will be completed using the principle of intention-to-treat [36]. We will include all randomized participants regardless of level of compliance with the protocol. The primary outcome variable is distance measured using the outcome of a 6MWT at 26 weeks.

Analysis of covariance will be used for this primary outcome, with treatment group as the main study factor and walking distance at baseline, weight, BMI, co-morbidities and patient preference as covariates. For participants with a missing outcome measure at 26 weeks, an imputation method will be used [37,38].

The secondary outcome variables include the 6MWT at 10 and 52 weeks post-surgery, the 15 m walk test, EQ5D, Oxford Knee Score, KOOS and knee ROM at most post-surgery time points.

Mixed model analyses will be used for the continuous variables measured repeatedly at 10, 26 and 52 weeks to estimate the treatment group by time effects. These analyses incorporate the missing data that may occur at the follow-up occasions. Baseline measurements of the outcome variables, together with factors such as weight, BMI, co-morbidities and patient preference, will be included as covariates [39]. For the binary outcome measured at 10, 26 and 52 weeks (knee flexion ≥100 degrees or ≤100 degrees), a Generalized Estimating Equation model will be used to test the treatment effect, with the adjustment of the covariates as above.

For the analyses involving the observational arm, the change score in the primary outcome will be compared between the observational group and those in the randomized home group adjusting for other covariates. A lack of significant difference in scores between the two groups will suggest there is no strong preference effect in this trial and we may not need to include preference as a covariate in the RCT analyses. To be consistent with the two RCT groups, we will aim for a minimum 64 participants in the observational group.

For the sensitivity analysis, the above analysis used for the primary and secondary outcome variables will be performed ‘as-treated’, analyzing patients according to the treatment they actually received and excluding those with missing outcome measures. It will only include patients who were compliant with the intervention protocol. Compliance for the HI group will be defined as attendance of a minimum of 7 days of inpatient rehabilitation, along with attendance at no less than two outpatient sessions. Compliance for the HO group will be defined as attendance at no less than two outpatient sessions. There will be procedures put in place to minimize loss to follow-up, such as obtaining multiple contact details at time of consent and reminders that assessments are due. The age, gender and height of participants will be used as stratification variables in the randomization procedure via a minimization algorithm. The mixed model analysis indicated above will include these three variables as additional covariates to incorporate the possible within-treatment group correlation associated with stratification [39].

### Trial organization

#### Ethical approval and trial status

Ethical approval has been granted by St. Vincent’s Human Research Ethics Committee for this trial (ethics reference: 11/125). Patient recruitment remained active at the time of submission of this protocol.

#### Trial co-ordination and trial progress

The HIHO trial team is listed in Table 3. The principal investigator will oversee the trial with the assistance of a co-ordinating investigator, and lead regular meetings of the field team and co-investigator group. The progress of the trial will be monitored and supported by the co-investigators. The principal and co-ordinating investigator will design the data collection forms.

**Table 3:** HIHO trial team

**Name**	**Role on team**	**Affiliation**
Dr. Justine Naylor	Principle investigator	Whitlam Orthopaedic Research Centre, South West Sydney Local Health District,
		University of New South Wales, Ingham Institute of Applied Medical Research
Mark Buhagiar	Co-ordinating investigator;	HammondCare, University of New South Wales,
	PhD candidate;	
	baseline assessor;	
	physiotherapist	
Professor Ian Harris	Co-investigator;	Whitlam Orthopaedic Research Centre, South West Sydney Local Health District,
	consultant orthopaedic surgeon	University of New South Wales, Ingham Institute of Applied Medical Research
Dr. Wei Xuan	Co-investigator;	Ingham Institute of Applied Medical Research
	senior biostatistician	
Associate Professor Friedbert Kohler	Co-investigator;	HammondCare, University of New South Wales, South West Sydney Local Health District
	consultant rehabilitation specialist	
Rachael Wright	Outcome assessor	South West Sydney Local Health District
Renee Fortunato	Outcome assessor	South West Sydney Local Health District
Danella Hackett	Physiotherapist;	South West Sydney Local Health District
	baseline assessor	
Dimyana Farag	Physiotherapist;	South West Sydney Local Health District
	baseline assessor	
HIHO: Hospital Inpatient versus HOme.		

The principal investigator will instigate and co-ordinate the training of the field team and perform audits of procedures throughout. The co-ordinating investigator will manage recruitment, data flow, recording and storage. Off protocol and adverse event reports will be sent to the data and safety monitoring board, with adverse events investigated as they occur, and reports monitored bimonthly. Adverse events will be independently reviewed by two members of the HIHO trial team, blind to participant arm allocation, with a third member to adjudicate in the event of disagreement.

#### Data and safety monitoring

An independent data safety monitoring board will be established to monitor the trial safety and, where appropriate, provide advice on issues regarding the scientific aspects of study conduct (eligibility, recruitment rates, compliance) and any emerging evidence as it relates to the trial. This board will comprise a rehabilitation physician, physiotherapist and a statistician/epidemiologist. The board will be notified of any adverse events (such post-operative complications that present after randomization or any falls whilst attending therapy) and will be required to decide whether they are related to the trial interventions. Reference to past incidents of falls during rehabilitation sessions, for example, will be used to guide such decisions. If there appears to be an atypical trend in adverse events, the trial will be suspended.

#### Intervention fidelity

Adherence to the intervention protocols will be facilitated by collaborative development of the protocol, protocol-based delivery, a comprehensive manual of standard operating procedures, training of involved personnel, structured recording forms, audit, observation and feedback.

#### Publication policy

Irrespective of outcome, the results of the trial will be submitted for publication in an appropriate journal. The principal investigator will be responsible for the compilation of report manuscripts, which the co-investigators will review and approve prior to submission. Any documents actuated from sub-studies arising from or related to the HIHO trial must be approved by the principal investigator. Other noteworthy contributors to the conduct of the HIHO Trial will be listed individually (Table 3) as an assemblage titled “the HIHO trial team”.

#### Timetable for the HIHO trial

May 2012 - Ethical approval granted by St. Vincent’s Human Research Ethics Committee

June 2012 - Recruitment starts

July 2012 - First participant randomized

July 2013 - First participant completes 1-year follow-up

December 2014 - Recruitment completed

March 2015 - Last participant randomized

March 2016 - Last participant completes 1-year follow-up

September 2016 - Analysis and publication of outcome data

## Discussion

The HIHO trial will be the first randomized trial to compare the effectiveness of inpatient rehabilitation with a hybrid home exercise program following a TKA for patients with knee OA. It will address the lack of randomized trials to assess post-surgical outcomes for commonly utilized treatment options. The results will help to establish the best rehabilitation approach for adults with moderate to severe OA of the knee undergoing TKA who are deemed sufficiently independent to be discharged directly home.

## Abbreviations

6MWT: 6-minute walk test; BMI: Body mass index; HI: Hospital inpatient rehabilitation; HIHO: Hospital inpatient versus hOme; HO: Hybrid home program; KOOS: Knee injury and osteoarthritis outcome score; OA: Osteoarthritis; RCT: Randomized controlled trial; ROM: Range of motion; TKA: Total knee arthroplasty; WJRC: Whitlam joint replacement centre.

## Competing interests

The authors declare that they have no competing interests.

## Authors’ contributions

MAB leads the co-ordination of the study, participated in the design of the study, and wrote the first draft of this manuscript. JMN conceived of the project, led the design of the study, and contributed to the manuscript. WX provided statistical advice and assisted with writing the relevant sections of the manuscript. IAH and FK participated in the design and co-ordination of the study. RF and RJW participated in the co-ordination and data acquisition of the study. All authors provided comments on drafts of this paper, and approved the final manuscript.
